# Obesity, Osteoarthritis, and Myokines: Balancing Weight Management Strategies, Myokine Regulation, and Muscle Health

**DOI:** 10.3390/nu16234231

**Published:** 2024-12-07

**Authors:** Daniel Vasile Timofte, Razvan Cosmin Tudor, Veronica Mocanu, Luminita Labusca

**Affiliations:** 1 Department of Surgery, “Grigore T. Popa” University of Medicine and Pharmacy, 16, Universitatii Street, 700115 Iasi, Romania; daniel.timofte@umfiasi.ro (D.V.T.); razvan.tudor@umfiasi.ro (R.C.T.); 2 Dr. Iacob Czihac Military Emergency Hospital Iasi, General Henri Mathias Berthelot Str. 7-9, 700483 Iași, Romania; 3 Department of Morpho-Functional Sciences II (Pathophysiology), Center for Obesity BioBehavioral Experimental Research, “Grigore T. Popa” University of Medicine and Pharmacy, 700115 Iasi, Romania; 4 Department of Orthopedics and Traumatology, “Sf. Spiridon” Emergency Clinical Hospital, 700111 Iasi, Romania; llabusca@phys-iasi.ro; 5 National Institute of Research and Development in Technical Physics Iasi, 700050 Iasi, Romania

**Keywords:** obesity, osteoarthritis, myokines, weight management, physical activity, inflammation

## Abstract

Obesity and osteoarthritis (OA) are increasingly prevalent conditions that are intricately linked, with each exacerbating the other’s pathogenesis and worsening patient outcomes. This review explores the dual impact of obesity on OA, highlighting the role of excessive weight in aggravating joint degeneration and the limitations OA imposes on physical activity, which further perpetuates obesity. The role of muscle tissue, particularly the release of myokines during physical activity, is examined in the context of OA and obesity. Myokines such as irisin, IL-6, and myostatin are discussed for their roles in metabolic regulation, inflammation, and tissue repair, offering insights into their potential therapeutic targets. This review emphasizes the importance of supervised weight management methods in parallel with muscle rehabilitation in improving joint health and metabolic balance. The potential for myokine modulation through targeted exercise and weight loss interventions to mitigate the adverse effects of obesity and OA is also discussed, suggesting avenues for future research and therapy development to reduce the burden of these chronic conditions.

## 1. Introduction

Obesity is a chronic, multifactorial, and complex condition characterized by excessive adiposity, which significantly impacts overall health and increases the risk of various complications. Obesity is a major increasing health threat worldwide, and its prevalence and incidence justify the categorizing it as an epidemic [1]. The increasing consistency with which it is spreading globally affects quality of life and threatens improvements in public health [2].

Obesity is associated with a wide array of health complications, including but not limited to cardiovascular diseases, type 2 diabetes, certain cancers, musculoskeletal disorders, and metabolic syndrome. From a public health and healthcare system standpoint, obesity is not merely a result of individual behavior but is influenced by a confluence of genetic, environmental, behavioral, and socio-economic factors. These include poor dietary habits, sedentary lifestyles, genetic predisposition, psychological conditions, and socio-economic determinants that limit access to healthy food and opportunities for physical activity.

Osteoarthritis (OA) is a multifactorial, potentially disabling joint degenerative disease that has displayed an impressive rise in incidence and prevalence in recent decades [3]. OA-related joint pain is associated with motor impairment, sleep disturbances, fatigue, depressed mood, and a decline in independence. In comparison to peers matched by age and sex, individuals with OA incur higher out-of-pocket healthcare expenditures and face significant costs attributable to lost productivity. A substantial proportion of individuals with OA (ranging from 59% to 87%) also have at least one comorbid chronic condition, with a notable prevalence of obesity and cardiometabolic disorders. OA can impair the capacity of individuals to engage in physical exercise and achieve weight loss, thereby increasing their risk of adverse health outcomes [4].

There is therefore a bilateral interdependence between obesity and OA; their concomitance intricately contributes to enhancing both pathogenic mechanisms, worsening outcomes and patient experiences. The association of obesity and OA is consistently increasing health expenditures as well as expenses for assisted living.

It has been stated that weight loss is one of the few interventions that can consistently contribute to both OA- and obesity-associated health-risk preventions. Single or associated methods of weight loss such as diet, exercise, obesity drugs, or bariatric surgery decrease pain even in non-weight bearing joints and fight motor disability associated with loss of joint function [5,6,7]. A recent meta-analysis, however, revealed that weight loss only modestly contributes to pain reduction, functional scores, and structural outcomes in hip and knee OA [8]. Rather, weight gain prevention, weight loss magnitude, weight management, and the choice of weight loss modality may impact OA occurrence and progression in obese individuals [9].

One important, yet constantly overlooked, factor influencing joint health is muscle tissue in general and the periarticular muscle compartment in particular. Muscle strength and muscle power [10] are important parameters in hip and knee OA primary and secondary prevention and management [11]. Muscle rehabilitation interventions are used to complement the effectiveness of symptomatic therapies [12], alignment, or total joint replacement surgery [13]. Muscle-dependent joint biomechanics has long been recognized and employed to support and correct articular structure and function [14]. Periarticular muscle joint disbalance around the hip and knee is known to strongly promote primary and/or secondary OA in respective joints [15]. Even though maintenance of biomechanical stability is crucial for normal joint functioning, recent insights point towards the systemic and endocrine role of muscle-released cytokines. The role of myokines in musculoskeletal tissue turnover, the immune system, and metabolic balance is currently recognized to go beyond mere biomechanics [16]. Myokines are increasingly recognized to play an important systemic role in maintaining health by regulating metabolic, inflammatory, and regeneration processes, involving a large diversity of body maintenance processes such as metabolic regulation, insulin sensitivity, fatty acid metabolism, endothelial function, angiogenesis, and neuroprotection. Dysregulated myokine activity has been associated with musculoskeletal degenerative diseases such as osteoporosis, sarcopenia, and cachexia [17] but also with systemic diseases such as insulin resistance, metabolic syndrome [18], obesity, autoimmunity, and Alzheimer’s [19,20]. Restoring muscle activity using physical training and exercise has been shown to positively influence the natural history of various musculoskeletal and systemic degenerative diseases, OA and obesity included [21,22]. We will focus on further describing myokines’ role in the maintenance of articular joint health. Current knowledge regarding myokine fluctuation during existing interventions for weight loss and weight management in obesity will be presented. The aim is to give an overview of existing knowledge on how weight loss methods affect myokine release and consecutively joint health, OA occurrence, progression, and management.

## 2. Myokines—The Currency of Physical Activity

A growing understanding has emerged regarding the role of the musculoskeletal system—bone, muscle, and fascia—in maintaining systemic balance through the regulation of electrolytes, signaling molecules, metabolism, and immune function [23]. Muscle tissue is increasingly recognized as a versatile organ with widespread functions throughout the body. The muscle compartment, as a whole, has a crucial role in supporting organisms’ integration within hierarchic ecosystems through body movement and consequent activities [24]. Both the skeletal muscle and smooth muscle components of this system contribute to the body’s adaptation to its environment from movement, and vascular activity to food intake, metabolism, and management of energy expenditure. As with bone and adipose tissue, muscle releases a plethora of small molecules that act in a paracrine and endocrine manner to harmonize function and contribute to prompt repair in many if not all body organs and systems. These signaling molecules have been given the generic name of myokines. “Myo-” (μυο-) is a prefix meaning “muscle” that originates from the Greek word “mys” (μῦς), “muscle” and “-kine” (κίνος) is a suffix meaning “movement” or “activity.” Together, the term “myokine” essentially translates to “muscle movement signaler,” reflecting the role of these molecules in mediating biological responses initiated by muscle activity [25]. Myokines are a broad category of cytokines, peptides, and proteins released by skeletal muscle cells in response to muscle contraction. Myokine signaling essentially accounts for correlating body functions with physical activity, justifying the inclusion of the skeletal muscle compartment in the category of endocrine organs. These signaling molecules play critical roles in autocrine, paracrine, and endocrine communication, influencing a wide range of physiological processes throughout the body, including metabolism, inflammation, and tissue repair [26]. Physical exercise has been recognized as a useful intervention for the prevention and treatment of metabolic degenerative diseases, from type 2 diabetes [27], obesity [28], and neurodegenerative diseases [29] to musculoskeletal tissue degenerative diseases including OA [30], tendinopathies [31], osteoporosis management, and prevention of falls [32]. Exercise therapy may benefit malignancies of various origins by modulating the immune response and preventing tumor-induced sarcopenia [33]. In this context, monitoring myokine release has been proposed as a modality for the follow-up of exercise-based interventions in these categories of patients [25,34]. Even though there are hundreds of molecules associated with documented or potential roles of myokine, in humans, only several of them have been related to a specific function, which will be briefly introduced in the following chapters.

Several myokines that have been identified so far are involved in a multitude of biochemical pathways, influencing body homeostasis, metabolism, inflammation, and disease progression [35]. Some myokines are predominantly produced by myocytes, while others, though not directly myocyte-derived, act as key mediators of pro- or anti-inflammatory responses, as well as trophic and regenerative effects, particularly during physical exercise [36]. Their currently known relations with physical activity, obesity, and osteoarthritis, as well as with muscle atrophy and sarcopenia, are introduced below.

## 3. Predominantly Skeletal Muscle-Specific Myokines

**Irisin** is produced by proteolytic cleavage of the membrane protein fibronectin type III domain-containing protein 5 (FNDC5) through PGC1-α (peroxisome proliferator-activated receptor-gamma coactivator-1 alpha) activation. PGC1-α is a transcriptional co-activator induced in skeletal muscles by exercise. Irisin is released within the bloodstream during exercise, having multiple systemic effects on metabolic regulation in terms of energy expenditure and insulin sensitization. Irisin precursor was able to promote differentiation “browning” of stromal vascular fraction cells derived from white adipose tissue in mice by increasing the expression of thermogenic genes such as UCP1 (Uncoupling Protein 1) [37]. Studies correlated irisin presence within the bloodstream with increased muscle metabolism, dependent on previous training and the intensity of physical activity. FNDC5 mRNA and bloodstream irisin levels were found to decrease after surgically induced weight loss together with decreasing body mass [38], pointing towards the necessity of investigating the effects weight loss methods have on the muscle compartment and metabolic body maintenance. Levels of circulating irisin were also found to be age-dependent, being higher in younger versus older subjects. However, increasing irisin levels were reported in healthy individuals as well as individuals with metabolic syndrome after a similar training regimen. Moreover, similar fluctuations in blood irisin levels could be observed in different age and fitness groups despite different starting basal levels [39]. Increased irisin levels are thought to be associated with increased skeletal myocyte glucose and lipid metabolism in a mechanism mediated by AMP-activated protein kinase (AMPK). AMPK acts as a metabolic sensor and key enzyme in muscle metabolism, and is probably the link between increased muscle activity, energy consumption, and signaling molecule release [40].

Serum irisin levels were found to be significantly increased compared to basal status after 12 weeks of endurance training in elderly (68 ± 8) compared to young (21 ± 1) adults, while reductions in visceral adipose tissue in elderly adults negatively correlated with irisin serum levels [41].

Other studies, however, found that mRNA levels of PGC-1α and FNDC5 in muscle biopsies of previously inactive individuals increased after 12 weeks of training while blood levels only increased modestly and shortly after exercise with few effects in increasing white adipose tissue (WAT) UCP1 [42]. Given the brief persistence within the bloodstream as well as the weak association of irisin and FNDC5 with glucose tolerance or with metabolic disturbances, the direct contribution of both the precursor and myokine on overall health might not be direct. It has been proposed that factors beyond PGC-1α and its transcription could influence FNDC5 expression and contribute to systemic metabolic effects [43].

It is important to note that not only skeletal myocytes but also WAT adipocytes have been found to release FNDC5, making it a possible myokine–adipokine signaling molecule. FDNC5 could be obtained from rat explants of WAT as well as from visceral adipose tissue and was expressed by the respective tissues in vivo after endurance training in rats [44]. Even though a similar dual role in humans has not been confirmed yet, irisin could be regarded as one of the factors involved in muscle–adipose tissue crosstalk. The situation of irisin release could, however, be different in obesity. In highly obese patients, irisin levels were found to be positively correlated with BMI and WAT mass. The existence of a mechanism of irisin resistance similar to that already described in the case of insulin and leptin could be a compensatory mechanism from one side and could threaten the efficiency of irisin as a therapeutic agent in such patients [45].

## 4. Irisin in Osteoarthritis Pathogenesis—Multiple Roles in Supporting Articular Joint Tissues

In mice under experimental conditions, irisin was found to be involved in articular tissue joint development. Irisin protected surgical- induced mice OA and human OA cartilage samples from degradation, and rescued chondrocyte metabolism in irisin knock-out mice. Interestingly, irisin expression could be found by immunohistochemistry in pre-hypertrophic and hypertrophic zones of growth plate cartilage and postnatal mice while being absent in the proliferating and resting areas of the same tissue [46]. Irisin-knocked-out mice developed severe OA while intraarticular delivery of recombinant irisin inhibited OA progression (OARSI histological score) assessed at 8 weeks. Translational and clinical evidence supports the fact that exercise-induced increases in irisin levels influence metabolic syndrome, attenuate aging, and protect articular joint tissue from OA development [47]. Resistance training in high-fat diet-fed prediabetic mice increased irisin levels and was associated with decreased serum cholesterol and triglycerides [48]. Not all studies are consistent in correlating exercise with increased irisin levels. The type and dynamics of serum irisin fluctuations could be some of the reasons for conflicting reports. A recent meta-analysis curated 560 human studies of which only 7 met the inclusion criteria and concluded that exercise increased irisin serum levels, ultimately affecting WAT metabolism and thermogenesis [49]. Skeletal muscle secreted irisin during exercise positively influences subchondral bone density and increases chondrocyte proliferation, therefore contributing to joint protective mechanisms. Irisin’s role in decreasing JNK phosphorylation levels with consecutive reductions in inflammatory markers, (IL-1), and the inhibition of chondrocyte catabolism observed in vitro [50] could also act in protecting senescence-induced OA progression.

By binding to transmembrane integrin receptors, irisin activates intracellular wnt/β-catenin and ERK/MAPK, influencing the balance between osteoblast and osteoclast activity within the subchondral bone, resulting in increased structural integrity and mechanical support in preclinical settings as well as in clinical studies [51]. Exercise increased irisin expression and ameliorated osteoporosis and osteoarthritis progression in estrogen-deficient rats (ovariectomized) [52], offering potential targets for treating estrogen deficiency-aggravated bone loss and OA.

Conversely, increased irisin expression inhibits Wnt/β-catenin and NF-κB activation in OA chondrocytes, partially restoring anabolic/catabolic activity, increasing autophagy and proliferation, and supporting cartilage extracellular matrix (ECM) [53]. Irisin’s role in mitigating inflammatory markers and chondrocyte pyroptosis has been proved in a preclinical study. Moderate-intensity training in rats increased blood and synovial fluid levels of irisin, recovering the expression of collagen II, and attenuated MMP-13 and ADAMTS-5 in vitro in IL-1β-induced chondrocytes [54]. Exercise-induced irisin and lubricin release can act on cartilage, influencing OA progression by inhibiting inflammation and supporting joint lubrication and functional biomechanics [55], both being valuable biomarkers as well as possible targets for OA. Moreover, as enhanced metabolism and autophagy in meniscal cells have been shown to prevent posttraumatic OA in experimental models [56], interventions such as exercise, quadriceps strengthening, and the associated release of irisin could serve as a preventative strategy for OA following joint trauma [57].

## 5. Irisin in Muscle Atrophy and Sarcopenia—Can It Function as a Predictive Biomarker?

In a prospective cross-sectional study including 715 subjects of both sexes, blood irisin levels positively correlated with appendicular lean mass and hand grip strength being lower in individuals with identified sarcopenia [58]. Decreased levels of irisin < 1.0 μg/mL for men and <1.16 μg/mL for women were proposed to be predictive for the onset of sarcopenia after adjusting for age and sex [59] with similar findings in postmenopausal women [60]. However, circulating irisin levels could not be associated with muscle mass and function in the elderly and may not be useful as a marker in the geriatric population [61]. Irisin release might be affected by age-related muscle atrophy. As expected, mice and human serum samples from older individuals were found to display decreased levels of circulating irisin mRNA levels while FDNC5 knock-out mice displayed aggravated muscle performance. Recombinant irisin administration was able to rescue muscle strength in both aged and irisin-deficient mice while decreasing fat tissue expansion, insulin resistance, and liver steatosis [62]. In another study, disease-related malnourishment and subsequent sarcopenia correlated well with decreased irisin levels after age and sex adjustments but not with myostatin levels, which remained similar in sarcopenic and non-sarcopenic subjects [63]. Decreased irisin serum levels and increased tumor necrosis α(TNFα) in newly diagnosticated patients with malignancies correlated with decreased muscle performance [64], possibly pointing towards an inverse correlation between systemic inflammation levels and irisin release, which could explain the lack of predictability of irisin in the case of older adults. Even more notable for the context of this work, serum irisin levels were found to be positively correlated with sarcopenic obesity in patients with type 2 diabetes [65]. Even though the correlation between sarcopenic obesity, irisin levels, and OA has not been examined, the outlined intricate pathogenic mechanism could prompt further investigation.

The association of various methods of weight loss and management with increased irisin levels is notable as it could correlate with improved articular joint pain, OA prevention, and/or management after such interventions. In bariatric surgery patients, increased irisin levels after nine months but not after one month correlated with the total amount of fat loss post-surgery. Body weight reduction following 6 months of diet, exercise, and behavioral cognitive therapy did not result in significant circulating irisin level changes; however, patients who displayed a significant increase had lower fasting insulin levels and insulin resistance [66] (Table 1). This suggests that irisin fluctuations may be influenced by individual factors and, in some cases, particularly after bariatric surgery, improvements in irisin levels may take longer to manifest.

β-aminoisobutyric acid (BAIBA) produced during the catabolism of thymine is released by skeletal muscle myocytes during physical activity. Metabolomics profiling of skeletal myocytes identified BAIBA as a myokine associated with muscle activity. BAIBA induced expression of brown adipocyte genes in WAT adipocytes and β-oxidation in hepatocytes in vitro and in vivo by a PPARα mechanism [67]. BAIBA has been identified as one of the thermogenetic factors involved in the browning of WAT together with physical exercise, irisin, gamma amino butyric acid (GABA), PPARɣ agonists, and JAK inhibitors. Browning of WAT and the possible therapeutic targets offered by browning agents could serve as important factors in the treatment and prevention of obesity and metabolic syndrome [68]. BAIBA stimulates the oxidation of fatty acids by reducing WAT-specific lipogenesis through an AMPK-mediated mechanism and is involved in mitigating inflammation and insulin resistance [69]. BAIBA was reported to reduce hyperlipidemia-induced endoplasmic reticulum stress and to protect hepatocytes from apoptosis. BAIBA reduces free fatty acid (FFA) β-oxidation, and ketone body production, as well as increasing mRNA of the β-oxidation-limiting enzyme carnitine palmitoyltransferase 1 (CPT-1). Its role in linking physical activity and metabolism by acting as a possible mediator of inter-tissue and organ communication during exercise could be further used as a target for treating metabolic syndrome and its complications [70]. While there is no available correlation between BAIBA fluctuation in OA models or clinical studies, its association with bone mineral density might stimulate further research. Enantiomer, as well as a gender-dependent correlation with mineral bone mass density, suggest that any prospective study should consider these parameters. L-BAIBA may be correlated with bone density and body composition in females but not in males while D-BALB could serve as a marker of aging and decreasing physical activity [71]. Experimental diet-induced obesity could be prevented by oral BAIBA administration in partially leptin-deficient mice. The accumulation of body fat as well as the onset of insulin intolerance, liver steatosis, and hypertriglyceridemia as well as leptin levels could be restored in partially but not completely leptin-deficient mice [72]. Relative to weight loss management, a low-caloric diet was found to increase BAIBA serum levels independent of physical activity in obese women. Increased BAIBA serum levels correlated with weight loss, improved energy metabolism, and insulin levels [73].

Regarding sarcopenia, BAIBA addition in the drinking water of mice in a mice model of osteocyte apoptosis protected them from bone and muscle loss. However, this effect was lost in the case of age-related sarcopenia due to a decrease in muscle Mas-related G protein-coupled receptor type D (mrgprd). Skeletal muscle mrgprd was found to mediate the protective effect of BAIBA osteocyte against ROS-related mitochondrial breakdown in mice. Its diminished expression might explain its reduced role in protection against bone loss in aging [74] (Table 2).

Existing evidence supports BAIBA involvement in reductions in fat mass, improvements in insulin sensitivity, and the promotion of the browning of adipose tissue. These findings suggest that BAIBA holds promise as a therapeutic agent for addressing obesity, insulin resistance, and potentially degenerative joint diseases. However, further research is necessary to test its importance as a targetable molecule for potential therapeutic applications.

Isoform 15 of the C1q/TNF-related protein (CTRP) family (CTRP15), also known as myonectin, is expressed by differentiated myotubes of skeletal muscle. CTRP15 mRNA transcripts as well as blood levels were found to increase upon voluntary exercise, decrease by fasting, and be restored by refeeding [75]. Compounds that increase cellular cAMP production were found to increase myonectin transcripts. Cultured adipocytes and hepatocytes treated with myonectin increased fatty acid uptake by cells and its administration to mice reduced the levels of circulating free fatty acids (FFAs). These findings lead to the conclusion that myonectin might serve as the connection between muscle activity and lipid metabolism within WAT and the liver [49]. Lower circulating levels of myonectin were found in obese, type 2 diabetic patients and patients with glucose intolerance [76], while its increase was proposed as a biomarker for the development of diabetes mellitus [77] or as a marker of metabolic syndrome [78]. Myostatin overexpression in these cases could be a compensatory mechanism for counteracting insulin resistance [79]. Of note, CTRP-15 has a similar structure to WAT-released adiponectin, an insulin-sensitizing adipokine with anti-diabetic and anti-inflammatory functions in mice and humans [80]. Other members of the large and ubiquitous C1q protein family with homology to the immune complement C1q (such as cerebellin, adiponectin, and collagen VIII and X) display a large variety of functions in development, growth, immunity, and metabolic balance [81].

An in vitro study showed that myonectin inhibited mouse osteoblast differentiation and alkaline phosphatase activity as well as osteoclast formation in bone marrow cells at least in part by inhibiting mitochondrial biogenesis [82]. A link between increasing serum myonectin levels in insulin resistance and metabolic syndrome as a compensatory mechanism and the impaired subchondral bone metabolism during OA warrants further investigation.

Of note, after the laparoscopic sleeve procedure for weight management increased, myonectin serum levels were found to be negatively correlated with weight, waist, and hip circumference, body mass index, fasting plasma glucose, and HbA1c up to 6 months after surgery [83]. Notably, the long-term effects of bariatric surgery that yielded good anthropometric results did not completely restore metabolic features compared to normal weight controls, while maintaining improvement compared to obese subjects, including lower myonectin levels [84].

As with irisin, skeletal muscle-released myonectin contributes to lipid metabolism via the cluster of differentiation 36 (CD36) gene, fatty acid transporter protein (FATP), and fatty acid binding protein (FABP4). FABP4, however, does not involve glucose balance and lipolysis. Stimulation of myonectin release by exercise as well as lipids and glucose increases fatty acid uptake and storage by the liver and WAT, while decreasing circulating FFAs. This mechanism is reportedly involved in the prevention of insulin resistance. However, the development of hyperlipidemia-induced skeletal muscle insulin resistance leads to reactive oxygen species accumulation and subsequent mitochondrial dysfunction, altering skeletal muscle metabolism [85]. In this situation, myonectin could further contribute to insulin resistance due to impaired muscle–adipose tissue cross-talk [86] (Table 3).

Myonectin treatment could restore skeletal muscle mass in aged as well as Duchenne dystrophy mice models, mitigating mitochondrial dysfunction and restoring the expression of mitochondrial biogenesis-associated genes including PGC1α [87]. Myonectin administration was found to protect cardiac muscle from ischemia–reperfusion injury [88]. In preclinical settings, myonectin protected mice via activation of AMPK/PGC1α signaling from skeletal muscle atrophy in several models (age-related, sciatic nerve denervation, or dexamethasone-induced). In addition, myonectin administration was shown to protect against accelerated muscle loss in accelerated aging models in mice and an mdx model of mice with Duchenne muscular dystrophy, pointing towards its role in supporting skeletal muscle metabolism [89]. There could be a place for using myonectin as a potential therapy for OA associated with cardiovascular and cardiometabolic diseases. A duration of 8 weeks of aerobic exercise training for weight loss in obese women was found to increase myonectin serum levels and decrease insulin resistance compared to controls [90], pointing towards its role in exerting physical activity-based modulation of metabolic syndrome.

Clinical studies could not identify a direct correlation between myonecti serum levels and age-related sarcopenia in older Asian adults regarding skeletal muscle mass, grip strength, gait speed, the chair stand test, or short physical performance battery (SPPB) score [91]. Lifestyle changes that favor physical activity and preservation of skeletal muscle myokine synthesis including myonectin could at least in part prevent T2D-related sarcopenia that increases with age [92] (Table 3).

Existing evidence supports the potential use of myonectin as a biomarker for the management of weight loss after bariatric surgery. Further studies are needed to establish its role in follow-ups regarding skeletal muscle condition during weight loss management regimens that are not centered on physical exercise, such as bariatric surgery and medication.

## 6. Signaling Molecules Functioning as Myokines

One member of the large family of fibroblast growth factors (FGFs), FGF-21, is considered a “metabolic regulator” and is described as a contributor to controlling glucose and lipid metabolism. FGF21 stimulates glucose uptake by adipocytes, inhibits liver production of glucose, and protects pancreatic beta cells from glucose toxicity [93]. FGF21 increases in human plasma as well as skeletal muscle during hyperinsulinemia, and is therefore considered a myokine involved in regulating circulating insulin levels [94]. Evidence regarding FGF21’s function as an exercise-released myokine has yielded inconclusive results mainly because the relevant investigation focused on serum FGF21 levels in relation to exercise and not on its presence and/or increased transcription within skeletal muscle. FGF21 serum levels increased significantly in previously sedentary women after 2 weeks of exercise [95]. After a single round of “acute” exercise (30 min treadmill at 50 or 80 VO max of healthy male volunteers), FGF21 serum levels increased. Similarly, serum levels as well as FGF21 gene expression were induced in the liver but not in the skeletal muscle or adipose tissue of mice [96]. Increased liver transcription of FGF21 correlated with the increased expression of peroxisome proliferator-activated receptor alpha (PPARα), a factor involved in the activation of lipolysis as well as activating transcription factor 4 (ATF4). ATF4 is involved in the activation of autophagy during mitochondrial stress and is considered a regulator of osteoblast functions and a mediator of neural-dependent regulation of bone mass. FGF21 function as a possible missing link in brain–bone metabolism is currently being investigated [97].

FGF21 was found to ameliorate senescence and apoptosis as well as ECM catabolism in vitro in tert-butyl hydroperoxide (TBHP)-stressed chondrocytes by mediating apoptosis. In a mice model of damaged medial meniscus (DMM), FGF21 administration alleviated OA as assessed by Rx and OARSI histological score [98]. In a mice model of rheumatoid arthritis, FGF21 administration was shown to exert an antioxidant effect, inhibiting nuclear factor kappa-light-chain-enhancer of activated B cells (NF-κB)-induced inflammation, suggesting possible similar activity in limiting OA flares [99]. No clinical data are available to date regarding the possible role of FGF21 in OA prevention or progress; however, the proposed role of FGF21 agonists in treating liver steatosis and other metabolic diseases might be relevant for OA of metabolic origin or OA with disease comorbidities [100].

In mice, the glucagon-like peptide-1 receptor (GLP-1R) agonist (GLP-1RA) liraglutide and FGF21 exert similar metabolic benefits. Liraglutide administration increased liver FGF21 expression independent of a high carbohydrate diet, while Fgf21 knockout (LivFgf21−/−) mice did not respond to treatment. The GLP-1RA-FG21 axis could be exploited further for weight loss [101]. A correlation between the possible increase in FGF21 during physical exercise and its relevance to weight loss mechanisms and potential practicability derived from it will need further attention.

In a group of older adults, serum levels of circulating FGF21 were found to positively correlate with sarcopenia determinants (grip strength, muscle mass). Wherever these findings indicate a catabolic role of FGF21 or an adaptation to reduced muscle activity, further investigation is needed [102]. In another study, FGF21 levels were found to negatively correlate with muscle strength but showed no correlation with muscle mass, potentially reflecting a response of liver-derived FGF21 to reduced muscle strength [103].

Several interleukins with intricate roles in immunity as well as tissue repair have been reported to be associated with muscle activity.

Interleukin-6 (IL-6) is a cytokine produced immediately and in large quantities during tissue injuries as well as infection; it induces acute phase response mechanisms, immune reactivity, and hematopoiesis. Its dysregulation is implicated in chronic inflammation as well as autoimmunity [104]. During intense muscle activity, high levels of IL-6 are recorded whenever liver glycogen levels are low. IL-6 increases in this situation induce lipolysis and/or fatty acid oxidation by means of AMPK pathway and/or PI3-kinase pathway activation, thereby increasing energy availability [105]. Muscle activity-induced increases in calcium ions induce MAPK and calcineurin activation, which leads to IL-6 production [106]. Increased IL-6 levels have been found in preclinical models as well as in humans after intensive training or endurance exercise (marathon) together with anti-inflammatory IL-10 [107]. Both IL-6 and IL-10 were expressed in cultured myocytes stimulated with calcium ionophores [108]. The paradoxical situation of IL-6 exerting either pro-inflammatory or anti-inflammatory effects seems to depend on its mode of signaling. IL-6 activity via the membrane-anchored receptor IL-6R (mIL-6R), so-called “classic signaling”, is activated by the acute-phase response, hematopoiesis, and homeostatic processes [109]. The pro-inflammatory function of IL-6 arises from trans-signaling when the soluble form of IL-6 receptor (sIL-6R) is generated through cleavage by sheddases such as ADAM10 and ADAM17. These enzymes are activated by the depletion of cholesterol or by phorbol 12-myristate 13-acetate (PMA), leading to the release of sIL-6R, which then binds to IL-6. This IL-6/sIL-6R complex subsequently interacts with gp130-expressing cells, initiating pro-inflammatory signaling. Conversely, the anti-inflammatory effect of IL-6 is mediated through classical signaling. While soluble glycoprotein130 (sgp130) exhibits low affinity for IL-6 alone, it demonstrates high affinity for the IL-6/sIL-6R complex (known as hyper-IL-6). Consequently, sgp130 can bind to this complex and inhibit the pro-inflammatory signaling pathway, thus exerting an anti-inflammatory effect [102]. IL-6 released during exercise has an anti-inflammatory effect, while leucocyte-derived IL-6 release exerts a pro-inflammatory effect possibly related to plasma levels of sIL-6 and sgp130. There might be variability between clinical observation results and the preexistent inflammatory and metabolic statuses of investigated subjects. Thus, in sedentary overweight males, moderate exercise increased IL-6 plasma levels while decreasing sIL-6 and sgp130 [110]. A single bout of exercise in children with juvenile arthritis resulted in increased plasma IL-6 and sgp130 levels, while sIL-6R concentration decreased [111]. Muscle-derived IL-6 might function not as an inflammatory cytokine but rather as an anti-inflammatory one. Summing up, the divergent roles of IL-6 are dependent on the signaling system, as well as on the activation of the sheddases ADAM10 and ADAM17. Additionally, the downstream signaling molecules transcribed in response to IL-6 vary depending on its concentration. It is possible that in individuals with pre-existent systemic inflammation, muscle IL-6 release has divergent effects.

In OA patients, increased serum and synovial fluid IL-6 levels have been correlated with disease progression and worsened prognosis. IL-6 activity results in ECM degradation via induction of MMPs (3,13), ADAMTS, matrix mineralization [112], and reduced chondrocyte proliferation. However, the role of IL-6 trans-signaling in cartilage degradation does not seem to be straightforward since soluble IL-6 was found to augment the production of anti-catabolic TIMPs in chondrocytes, pointing toward a cartilage-protective role [113]. The expression levels of IL-6 receptors in chondrocytes, and potentially in other articular cell types such as synovial fibroblasts, may play a crucial role in determining the balance between IL-6’s pro-catabolic and anti-catabolic effects.

The time-dependent immediate increase in IL-6 serum levels after physical exercise might function as an acute serum response to muscle stress that induces classical IL-6 signaling, harnessing its anti-inflammatory effects. In tumor-bearing mice, acute (up to a 29-fold increase) IL-6 skeletal muscle levels led to a significant decrease in tumor volume and an increase in natural killer (NK) cells infiltrating the tumor. Since the effect was reduced by the administration of IL-6 antagonists, the authors concluded that, at least in part, IL-6 mediated the acute serum response after skeletal muscle activity prompted NK cells to infiltrate tumors and to exert their specific clearing activity [114].

In a small patient group, early weight loss after sleeve gastrectomy was associated with a decrease in subcutaneous adipocyte size and decreased IL-6 mRNA levels [115]. Animal studies have pointed out that IL-6 overexpression was able to reduce weight gain while IL-6 deficient mice developed obesity at maturity [116]. As one of the acute phase response reactants, IL-6 as well as other inflammatory cytokines (such as TNFα) act in decreasing appetite and food intake. However, in the context of obesity and metabolic syndrome, chronic systemic inflammation is associated with the production of inflammatory cytokines by adipose tissue, partially reversed by weight loss and changes in lifestyle [117]. A systematic review and meta-analysis of ten studies involving 1537 patients revealed that a common IL-6 pathway inhibitor used as therapy for immune diseases such as rheumatoid arthritis (tocilizumab) was associated with an increase in body weight as determined by BMI, pointing towards a role of IL-6 signaling in weight management [118].

Exercise duration and intensity, diet and previous training levels, and particularly the preexistent glycogen levels within muscle consistently affect the level of IL-6 release. When released by muscle activity, Il-6 functions locally, systemically, and at the central nervous system levels (hypothalamus–pituitary axis) to maintain muscle fiber homeostasis during and following exercise. IL-6 activity contributes to exercise-associated health benefits, particularly in the context of chronic inflammatory diseases related to physical inactivity, such as type 2 diabetes, by increasing energy substrate availability, inducing fatigue to prevent excessive homeostasis stress, and promoting an anti-inflammatory environment within the bloodstream and tissues. Resistance training appears to be the most efficient in this respect, potentially reversing trans to cis signaling and promoting IL-6’s anti-inflammatory effect [119]. The relevance of this effect in the context of OA needs to be further investigated.

Perhaps not surprisingly, IL-6, as an inflammatory marker, has been associated with sarcopenia and cachexia in clinical studies as well as in animal models. Prospective clinical investigations have pointed out that elevated serum IL-6 could be associated with sarcopenia in elderly patients with hepatic cirrhosis [120] or chronic pulmonary obstructive disease (COPD) [121]. Increased IL-6 as well as IL-6/IL-10 serum levels were positively associated with degree of sarcopenia and increased with age—overall indicative of a systemic inflammatory status that cannot be compensated by anti-inflammatory IL-10 release [122]. Neoplastic-associated sarcopenia was found to correlate with increased IL-6 and CRP levels in post-surgical colorectal cancer patients [123]. These studies have not investigated the origin or signaling pathway of IL-6. In a mice C57BL/6 model of cancer-associated cachexia, increased serum levels of IL-6 were found to correlate with the inset of muscle and adipose waste. In this model, adipose tissue waste was caused by reduced lipid uptake and synthesis and increased lipolysis and not with increased elevated beta-adrenergic signaling or with the browning of adipose tissue. Muscle atrophy was reflected by decreased myofiber cross-sectional area. Changes were completely reversible after IL-6 was deleted from the injected cancer cells, pointing towards a causative role of IL-6 in the installation of neoplastic cachectic syndrome at least in this mice model [124]. Chronic inflammation associated with sarcopenia and sarcopenic obesity as well as aging (the latter commonly referred to as inflammation) converges in altering the immune profile of muscle components [125] with a tendency to release pro-inflammatory mediators such as IL-6. In turn, the inflammatory milieu might aggravate sarcopenia by activating the ubiquitin–protease system [126] and by antagonizing the muscle trophic role of insulin growth factor 1 (IGF-1) [127]. Chronic inflammation associated with low-level inflammatory cytokine release at the systemic and local level during obesity and aging may cause both insulin resistance (IR) as well as so-called anabolic resistance (AR). AR, defined as the impaired production of structural protein by skeletal muscle as a response to protein intake and physical exercise, is an important determinant of sarcopenia both in obesity and during aging. Healthy elderly require increased dietary protein intake compared to younger subjects to maintain similar myofibrillar protein synthesis {MPS) [128] and double the volume of physical exercise [129]. The synergistic effect of IR and AR can contribute to both age-related as well as obesity-related sarcopenia by mitochondrial disturbances, impaired protein synthesis, and myokine release that affects adipose tissue, skeletal muscle compartments, bone, and vascularization [130]. Efforts have been made to summarize the current data regarding the impact of sarcopenia, sarcopenic obesity, and circulating markers of inflammation in OA onset and progression [131] (Table 4).

IL-6, a key cytokine involved in both pro-inflammatory and anti-inflammatory processes, plays a dual role in influencing myokine activity and subsequent effects on joint health. During muscle activity, IL-6 is released in response to calcium-mediated signaling and acts through classical (cis) signaling pathways to exert anti-inflammatory effects. However, in the context of chronic systemic inflammation, as seen in obesity, IL-6 predominantly engages in trans-signaling via its soluble receptor (sIL-6R), promoting catabolic and inflammatory effects in joint tissues. This shift in IL-6 signaling has been implicated in matrix degradation and impaired chondrocyte function, which are key features of OA progression.

Current evidence exists to support the fact that the inflammatory milieu in obesity, characterized by elevated levels of TNF-α and other adipokines, exacerbates dysregulation of myokines such as myostatin and irisin. These inflammation-driven alterations in the myokine profile contribute to a vicious cycle of joint tissue degeneration, muscle weakness, and metabolic imbalances. The interplay between IL-6 signaling and myokines highlights the need for interventions that not only have the ability to reduce systemic inflammation but also can enhance the release of beneficial myokines through tailored physical activity. This dual approach could mitigate OA progression and improve metabolic health in individuals with obesity.

Interleukine-15 (IL-15) is another cytokine that has important functions in immune modulation released by skeletal muscle. IL-15 might function in an endocrine manner to adapt energy metabolism to physical activity, potentially acting in reducing adiposity and improving insulin sensitivity [132]. Resistance training in healthy individuals was found to double mRNA IL-15 levels in skeletal muscle even 24 h after training in a fiber muscle type-dependent manner [133]. IL-15 has been proposed as a major component of muscle–adipose tissue crosstalk in both animal models and human clinical studies. Mice overexpressing mIL-15 plasmids were able to run to exhaustion twice as long as controls. Fast-acting skeletal muscle fibers were found to overexpress sirtuin 1, peroxisome proliferator-activated receptor (PPAR)-δ, PPAR-γ coactivator-1α, and PPAR-γ coactivator-1β, subsequently increasing myosin heavy-chain and troponin I mRNA expression. Such findings indicate an increased oxidative phenotype as compared to controls, demonstrating a role for IL-15 in muscle adaptation to physical training [134]. Furthermore, IL-15 overexpression in mice was found to maintain insulin sensitivity during diet-induced obesity, maintaining lean body mass and low levels of visceral adiposity. The skeletal muscle of IL-15 overexpressing mice displayed increased mRNA levels of troponin1 as well as SRT1. SRT4 and UCP2 demonstrated increased oxidative metabolism [135].

Remarkably, IL-15 was able to induce hypertrophy in mature cultured human skeletal muscle cells [136]. IL-15’s ability to induce skeletal muscle hypertrophy by increased protein synthesis could be relevant for treating sarcopenia and cachectic syndromes without the risk of inducing uncontrolled cellular proliferative events [137]. Mice overexpressing IL-15 increased in lean body mass on both low-caloric and high-caloric diets, demonstrating a role of circulating (but not of locally released) IL-15 in regulating body fat composition [138]. A recent meta-analysis including 27 studies with 1310 participants undergoing acute or chronic physical exercise confirmed that acute exercise increases IL-15 levels one hour after training, with possible roles in improving metabolism in human adult subjects with different levels of prior training [139]. To date, no correlation between activity-induced muscle release of IL-15 and OA has been made. Given IL-15’s proposed role in attenuating age-induced skin deterioration via a muscle-regulated AMPK mechanism [140], its role in mitigating articular joint tissue deterioration could offer an avenue for further research (Table 5).

Members of the large transforming growth factor superfamily (TGF) were among the first isolated from skeletal muscle and described as signaling molecules involved in cell growth, differentiation, and apoptosis, as well as in muscle tissue regeneration and/or fibrosis [141].

One of the members of the TGF family, **myostatin** (also known as growth differentiation factor 8—GDF8), is expressed during the embryonic stage, and acts in limiting muscle growth during development [142]. **Myostatin’s** role in negatively regulating skeletal muscle growth continues in adulthood, having a role in balancing the anabolic/catabolic protein synthesis in skeletal muscle fibers. Several modalities for its targeting and inhibiting myostatin gene expression or receptors have been proposed as a therapeutic modality to counteract muscle atrophy of various causes (such as genetically inherited muscular dystrophies, aging, or AIDS–HIV-related) [143]. Myostatin circulates systemically in a latent form and becomes active upon enzymatic cleavage. Once activated, it binds with high affinity to ACTRIIB Activin receptors [144]. The consecutive activation of Smad family transcription factors, specifically Smad2 and Smad3, leads to muscle atrophy by subsequently activating the Forkhead Box family transcription factors, FOXO (1, 2, and 3), and inhibition of the AKT/mTOR pathway [145,146]. Increased levels of myostatin in patients with heart failure-driven muscle unloading [147] or cancer-related cachexia [148] seem to confirm its role as a negative skeletal muscle regulator. Conversely, the genetic deletion of the myostatin gene in vitro and from cardiomyocytes of mice models of heart failure prevented loss of skeletal muscle fibers, weakness, and exercise intolerance. In the same model, cardiac myostatin overexpression was found to increase blood levels of myostatin by four-fold with a consecutive reduction in skeletal and cardiac muscle weight [149]. Peroxisome proliferator-activated receptor gamma co-activator 1-alpha (PGC-1α) is stimulated by physical exercise and its upregulation increases muscle mitochondrial biogenesis. Compounds that target and inhibit myostatin increase PGC-1α, having FOXO inhibition and increased insulin sensitivity as a result [150] (Table 6).

White adipose tissue is another postnatal organ that expresses myostatin. Adipose tissue myostatin prevents the browning of adipose tissue and decreases insulin sensitivity. Increased mRNA myostatin and ActRIIb could be retrieved in visceral and subcutaneous fat of mice models of obesity [151] as well as in non-diabetic obese patients [152].

Weight loss management in a group of healthy obese patients consisting of physical exercise, diet, and counseling resulted in increased serum levels of both myostatin and adiponectin. However, the relative ratio between myostatin and adiponectin serum levels was dependent on the degree of weight loss; only subjects with more than 5% weight loss displayed increased strength and exercise capability. The authors concluded that monitoring the myostatin/adiponectin ratio during weight loss management could prevent skeletal muscle mass loss during such interventions [153].

Transgenic myostatin knockout mice displayed increased skeletal muscle mass compared to wild-type mice even when fed a high-fat diet. They also displayed increased expression of AMPK and phosphorylation within skeletal muscle with consecutive activation of PGC1α and Fndc5 (irisin precursor), demonstrating that targeting myostatin could be effective in treating obesity and metabolic syndrome [154].

No direct correlation has been established between physical activity and serum myostatin levels in animal models or humans. However, myostatin may function as an energy modulator in obesity, as studies have shown that trained obese mice exhibit increased ACTRIIb expression in brown adipose tissue compared to sedentary mice, while no such increase was observed in visceral fat [155].

Compared to healthy controls, myostatin serum concentration was found to increase in 184 patients with knee OA correlated with radiographic grading. More advanced OA stages (KL4) were found to exhibit increased myostatin serum levels compared to KL 3, 2. Myostatin (GDF8) presence within patients with an anterior cruciate ligament (ACL) tear was found to be predictive of periarticular bone resorption, muscle atrophy, and weakness at 6 months following ACL reconstruction procedures [156]. In a mice model of ACL tears, anti-GDF8 administration attenuated posttraumatic OA occurrence and periarticular bone loss while genetic deletion prevented bone and muscle deficiency after ACL transection in mice, suggesting that blocking myostatin can function as a modality to prevent posttraumatic OA [157].

Remarkably, myostatin was shown to promote macrophage type 1 (M1) polarization within nervous tissue (dorsal root ganglia), this being a key ligand in the neuroimmune connection that results in persistent OA pain. Circulating myostatin may be a cause for persistent joint pain even after the surgical removal of pain-producing tissues during total joint arthroplasty, particularly in the knee joint [158].

A retrospective study including muscle biopsy from patients who had undergone total hip arthroplasty (THA) for either femoral head fractures (subjects diagnosed with osteoporosis—OP) or patients who underwent THA for OA. Histomorphometry and immunohistology analysis displayed a decrease in muscle regeneration ability with decreased BMP2/4 and -7 expression and increased myostatin in OP patients compared to OA and healthy controls while OA patients displayed a higher number of regenerative Pax7 and myogenin-positive muscle fibers. The authors suggested that BMP and myostatin pathways are important for the induction of sarcopenia in OA and OP patients and could serve as therapeutic targets [159].

In a mice model of OA, hypergravity obtained by periodic centrifugation as a replacement for physical activity was found to prevent muscle fiber-type shifts and muscle loss, along with increases in both catabolic (myostatin and myostatin receptor) as well as irisin precursor gene expression. Hypergravity can mimic some of the effects of resistance training for trabecular bone and muscle mass but not for cortical bone maintenance [160].

Myostatin and its activin receptors (together with lipocalin A and growth differentiation factor GDF15) have been described as mediators of cachexia–anorexia syndrome associated with malignancies [161] together with circulating inflammatory factors. Targeting the myostatin pathway potentially offers an approach for treating cancer as well as age-, obesity-, and spinal dystrophy-related muscle loss associated with progressive body wasting and loss of motor function [162] (Table 6).

Folliculin (a Birt–Hoge–Dubé syndrome tumor suppressor) is a metabolic regulator involved in intracellular lysosomal nutrient distribution, a part of the signaling cascade that correlates nutrient availability with mTORC1 activation [163]. Folliculin has been related to skeletal muscle fiber specification and adaptation to training. Skeletal muscle fibers in mammals contain a variety of distinct types, which differ based on the type of myosin heavy chain (MHC) protein, number of mitochondria, and blood capillaries, and consecutively differ in fatigue resistance and metabolic energetic pathway [164]. Type I fibers (“slow twitch”) have a different type of MHC content and capillaries and support sustained physical activity that relies on oxidative phosphorylation. Type II “fast” fibers have less myoglobin content and rely on anaerobic glycolysis. They account for muscle strength and contraction speed but only for small spikes in activity and are quickly fatigued. Type IIa and IIx have intermediate characteristics between type I and II. Type I fibers rely on fatty acid oxidation for producing energy; therefore, increasing their representation within skeletal muscle has been regarded as a strategy to prevent obesity and metabolic-related diseases [165]. Folliculin-interacting protein-1 (FNIP-1) interacted with folliculin to control muscle fiber specification in mice, increasing type I fibers, muscle oxidative phosphorylation, and endurance [166]. FNIP-1, a key regulator of a mitochondrial function, might act through AMPK and mTORC1 activation in several other immune and metabolic functions such as the development of immune B and natural killer T cells (NKTs), muscle fiber-type specification, and browning of white adipocytes [167]. In mice, FNIP-1 activation using the AMPK pathway governed mitochondrial electron transfer chain complex assembly, fuel utilization, and, in consequence, exercise performance [168]. FNP-1 involvement in OA onset and progression has not been studied; however, evidence regarding the muscle–bone homeostatic axis via FNP-1-IGF2 in humans and mice might warrant further investigation [169].

## 7. Obesity, Weight Loss Management, and Musculoskeletal Health: A Proposed Road Ahead

**Figure 1:**
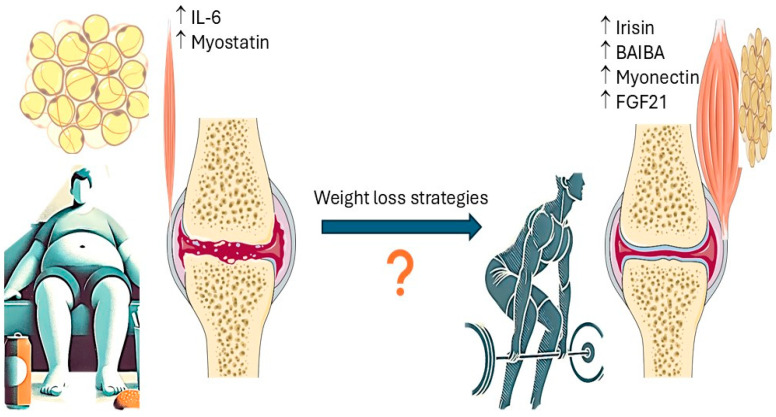
Sedentary life and obesity are associated with muscle inactivity/waste and predominance of pro-inflammatory status and catabolic myokines (such as Il-6 and myostatin), favoring occurrence and progression of osteoarthritis. Lean mass, brown adipose tissue, and physical activity increase predominance of anabolic myokines (such as irisin, BAIBA, myonectin, and FGF21) contributing to joint health. Ascending arrows signify increased myokine/cytokine expression. More evidence is needed to understand impact of different types of weight management strategies including physical activity on muscle mass, myokine release, and articular joint and overall health.

The intricate role of diseases centered on nutritional status and metabolic disturbances in the onset and/or aggravation of musculoskeletal diseases is increasingly recognized. Obesity and metabolic syndrome as well as cachexia have been correlated, and occasionally causally related to degenerative joint diseases in general and OA in particular. Regarding weight loss and particularly weight loss management, the importance of avoiding muscle atrophy and even sarcopenia, which sometimes accompany interventions, is recognized as important. Recent evidence supports the role of several weight loss strategies in alleviating symptoms and possibly acting in a disease-modifying manner concerning OA treatment. Thus, GLP-1 agonists were found to significantly reduce OA-related pain in obese individuals [170]. Little is known, however, about how weight loss surgery and medication impact skeletal muscle health, especially when interventions are not accompanied by physical exercise (Figure 1).

Physical activity and exercise are considered a crucial link between weight loss management and articular joint functional preservation. In this context, skeletal muscle as an endocrine organ involved in systemic metabolic balance is increasingly being seen as a therapeutic modality to address obesity, metabolic syndrome, and OA. There is, however, little existing evidence to support and inform physical therapy as a therapeutic regimen. In this respect, we propose that developing personalized exercise programs to optimize the release of specific myokines, such as irisin and IL-6, could be tested to enhance joint health and reduce systemic inflammation. Integrating myokine modulation into multidisciplinary weight management strategies that combine dietary, pharmacological, and physical activity interventions offers a comprehensive approach. Circulating myokine levels could serve as valuable biomarkers for tailoring and monitoring treatment efficacy in patients with OA and obesity. Additionally, promoting physical activity campaigns that emphasize the systemic benefits of exercise-induced myokine release could extend beyond weight management, highlighting broader public health benefits.

Thus, the complex interplay between obesity and osteoarthritis (OA) highlights the importance of addressing both conditions to improve patient outcomes. Obesity exacerbates OA through mechanical stress and systemic inflammation, while OA symptoms frequently limit physical activity. Pain-induced inactivity further contributes to weight gain and metabolic complications. Effective management strategies should encompass weight loss and muscle rehabilitation to enhance joint health and overall metabolic balance.

Recent research underscores the significant role of myokines, cytokines released by muscle tissue during physical activity, in maintaining systemic health. These molecules influence various physiological processes, including metabolism, inflammation, and tissue repair, which are crucial in the context of both obesity and OA. Notably, myokines like irisin, IL-6, and myostatin are implicated in muscle and joint health, with potential therapeutic implications for metabolic and musculoskeletal diseases.

Exercise-induced myokines promote beneficial adaptations in muscle and adipose tissues, supporting metabolic regulation and reducing inflammation. For instance, irisin facilitates energy expenditure and insulin sensitivity, while IL-6 modulates inflammation and energy substrate availability. Myostatin, on the other hand, negatively regulates muscle growth, but its inhibition may offer therapeutic potential in preventing muscle atrophy and enhancing metabolic health.

A significant limitation in the current body of research is the lack of standardized methodologies for measuring myokine activity across diverse populations and intervention studies. Variations in techniques, inconsistent sampling protocols, and different analytical approaches have led to discrepancies in the reported findings, which appear contradictory. This situation challenges our ability to draw definitive conclusions that can inform clinical practice as well as future research. Standardized frameworks for assessing myokine levels, including the timing of measurements relative to exercise or interventions, the selection of biomarkers, and population-specific factors such as age, sex, and metabolic status, are urgently needed. Furthermore, there is a pressing need for well-designed clinical trials that evaluate the efficacy of exercise regimens tailored to modulate myokines in both OA and obesity. Such trials should incorporate robust outcome measures, including joint health parameters, systemic inflammation markers, and functional performance indices, to elucidate the therapeutic potential of myokine-targeted strategies and inform clinical practice.

Addressing the bilateral relationship between obesity and OA through integrated approaches involving weight management, physical activity, and myokine modulation could provide extensive benefits. Future research should focus on elucidating the precise mechanisms by which myokines influence joint and metabolic health, optimizing exercise regimens to enhance myokine release, and developing targeted therapies to mitigate the adverse effects of obesity and OA. Myokines could be used as biomarkers for following up on different weight loss strategies as well as overall health status or could be targeted for pharmacological intervention.

This integrative approach could significantly reduce the burden of these chronic conditions and improve the quality of life and promote the long-term health of affected individuals.

## Figures and Tables

**Table 1:** Summary of the metabolic role of irisin and its impact on osteoarthritis, weight loss, and sarcopenia.

Irisin	Details
Source and Activation	Irisin is produced by cleavage of FNDC5, induced by PGC1-α activation during exercise within skeletal muscle
Metabolic Effects	Regulates energy expenditure, promotes insulin sensitization, and induces “browning” of white adipose tissue
Association with Exercise	Irisin levels increase with exercise, correlating with increased energy expenditure and browning of adipose tissue in animal models and possibly in humans
Age-Related Differences	Higher levels are found in younger individuals and levels increase with training in all age groups
Effects on Osteoarthritis	Protects against cartilage degradation, increases bone density, and reduces inflammation in OA models
Impact on Sarcopenia	Lower irisin levels associated with sarcopenia, may serve as a biomarker for muscle atrophy
Impact of Weight Loss	Irisin levels increase post-bariatric surgery, display correlation with fat loss and lower insulin levels and insulin resistance
Potential Biomarker Role	Could serve as biomarker for metabolic health, OA progression, and muscle conditions like sarcopenia

**Table 2:** Summary of metabolic role of BAIBA and its impact on osteoarthritis, weight loss, and sarcopenia.

BAIBA	Details
Source	Produced during catabolism of thymine, released by skeletal muscle myocytes during physical activity
Mechanism of Action	Induces browning of white adipose tissue (WAT) and β-oxidation in hepatocytes via PPARα mechanism
Metabolic Effects	Reduces WAT-specific lipogenesis, mitigates inflammation and insulin resistance, and enhances FFA oxidation
Protective Roles	Protects hepatocytes from apoptosis, reduces hyperlipidemia-induced stress, may protect against bone and muscle loss in sarcopenia
Potential Therapeutic Uses	Potential treatment for obesity, metabolic syndrome, and sarcopenia; may correlate with bone density
Gender-Dependent Effects	L-BAIBA may be linked to bone density and body composition in females, not males; D-BAIBA could serve as marker of aging
Impact on Obesity	Prevents diet-induced obesity and associated metabolic disorders in mice
Impact on Sarcopenia	BAIBA addition protects against bone and muscle loss in animal models, though effects diminish with age-related sarcopenia

**Table 3:** Summary of the metabolic role of myonectin and its impact on osteoarthritis, weight loss, and sarcopenia.

Myonectin	Details
Expression and Regulation	Expressed by differentiating myotubes in skeletal muscle; increased with exercise, decreased by fasting, restored by refeeding
Lipid Metabolism	Increases fatty acid uptake in adipocytes and hepatocytes; reduces circulating free fatty acids (FFAs)
Role in Metabolic Diseases	Lower levels in obese, type 2 diabetic patients; potential biomarker for diabetes mellitus and metabolic syndrome
Comparison to Adiponectin	Shares structural similarities with insulin-sensitizing adipokine adiponectin
Impact on Bone and Muscle Health	Inhibits osteoblast differentiation and osteoclast formation; linked to subchondral bone metabolism
Cardiometabolic Protection	Protects against skeletal muscle atrophy, cardiac muscle ischemia, and metabolic dysfunctions
Response to Weight Management	Increased after weight loss interventions; associated with improvements in insulin resistance
Impact on Sarcopenia	No direct correlation with age-related sarcopenia was observed in some studies

**Table 4:** Summary of the systemic role of IL-6 and its impact on osteoarthritis, weight loss, and sarcopenia.

IL-6	Details
Cytokine Type	Pro-inflammatory cytokine produced during tissue injury, infection, and muscle activity.
Acute Phase Response	Induces immune reactivity, hematopoiesis, and acute phase response mechanisms.
Metabolic Regulation	Promotes lipolysis and fatty acid oxidation during muscle activity; contributes to energy availability.
Signaling Pathways	It could exert pro-inflammatory or anti-inflammatory effects depending on the signaling pathway (classic vs. trans-signaling).
Exercise Impact	IL-6 levels increase during intense exercise, potentially offering anti-inflammatory benefits and contributing to muscle fiber homeostasis.
Role in Osteoarthritis (OA)	Elevated in OA, associated with disease progression, extracellular matrix (ECM) degradation, and altered chondrocyte function.
Impact on Sarcopenia and Cachexia	Associated with increased sarcopenia and cachexia, particularly in conditions like chronic inflammation, cancer, and aging.
Obesity and Metabolic Syndrome	Chronic IL-6 release in obesity contributes to systemic inflammation; implicated in appetite regulation and potential weight management.
Therapeutic Implications	IL-6 pathway inhibitors used in conditions like rheumatoid arthritis; effects on weight and inflammation are of interest for broader applications.

**Table 5:** Summary of IL-15’s role in obesity and osteoarthritis.

Activity	Details
Lean Body Mass Regulation	Overexpression in mice increases lean body mass across diets, demonstrating IL-15’s systemic role in regulating fat composition.
Effects of Exercise	Meta-analysis indicates acute exercise significantly increases IL-15 levels, enhancing metabolism across varying fitness levels.
Potential in OA and Skin Health	Potential to mitigate articular joint tissue deterioration and age-related skin issues through AMPK-regulated mechanisms.

**Table 6:** Summary of the metabolic role of myostatin and its impact on osteoarthritis, weight loss, and sarcopenia.

Myostatin	Details
Function	Negatively regulates skeletal muscle growth, balancing anabolic/catabolic protein synthesis.
Expression	Expressed during the embryonic stage; continues to regulate muscle growth in adulthood.
Receptors	Binds to ACTRIIB Activin receptors, activating Smad2/3, leading to muscle atrophy.
Cachexia and sarcopenic obesity	Increased levels associated with muscle wasting in heart failure, cancer cachexia, and obesity.
Therapeutic Target	Inhibiting myostatin is explored as a treatment for muscle atrophy, obesity, and metabolic syndrome.
Impact on Adipose Tissue	Prevents the browning of white adipose tissue (WAT), decreases insulin sensitivity, and may contribute to obesity-related metabolic issues.
Osteoarthritis (OA) Connection	Higher myostatin levels in OA linked to disease severity, muscle atrophy, and bone resorption; potential target for preventing posttraumatic OA.
Role in Muscle and Bone Maintenance	Myostatin inhibition shown to improve muscle mass, and bone density, and prevent muscle fiber shift in various models, including hypergravity simulation.
Impact on Pain	Promotes M1 polarization in nervous tissue, potentially contributing to persistent pain in OA and after joint surgery.
Therapeutic Potential	Targeting myostatin could offer treatment approaches for sarcopenia, cancer cachexia, and OA-related muscle wasting and bone degeneration.

## References

[1] (2000). Obesity: Preventing and Managing the Global Epidemic.

[2] Mehta N.K. (2023). Obesity as a Main Threat to Future Improvements in Population Health: Policy Opportunities and Challenges. Milbank Q..

[3] Hawker G.A. (2019). Osteoarthritis is a serious disease. Clin. Exp. Rheumatol..

[4] Leifer V.P., Katz J.N., Losina E. (2022). The burden of OA-health services and economics. Osteoarthr. Cartil..

[5] Beavers D.P., Beavers K.M., Loeser R.F., Walton N.R., Lyles M.F., Nicklas B.J., Shapses S.A., Newman J.J., Messier S.P. (2014). The independent and combined effects of intensive weight loss and exercise training on bone mineral density in overweight and obese older adults with osteoarthritis. Osteoarthr. Cartil..

[6] Pinzariu A.C., Oboroceanu T., Eloae F.Z., Hristov I., Costan V.V., Labusca L., Cianga P., Verestiuc L., Hanganu B., Crauciuc D.V. (2018). Vitamin D as a Regulator of Adipocyte Differentiation Effects in vivo and in vitro. Rev. Chim..

[7] Vincent H.K., Heywood K., Connelly J., Hurley R.W. (2012). Obesity and weight loss in the treatment and prevention of osteoarthritis. PM R.

[8] Panunzi S., Maltese S., De Gaetano A., Capristo E., Bornstein S.R., Mingrone G. (2021). Comparative efficacy of different weight loss treatments on knee osteoarthritis: A network meta-analysis. Obes. Rev..

[9] Lim Y.Z., Wong J., Hussain S.M., Estee M.M., Zolio L., Page M.J., Harrison C.L., Wluka A.E., Wang Y., Cicuttini F.M. (2022). Recommendations for weight management in osteoarthritis: A systematic review of clinical practice guidelines. Osteoarthr. Cart. Open.

[10] Reid K.F., Price L.L., Harvey W.F., Driban J.B., Hau C., Fielding R.A., Wang C. (2015). Muscle Power Is an Independent Determinant of Pain and Quality of Life in Knee Osteoarthritis. Arthritis Rheumatol..

[11] Bennell K.L., Hunt M.A., Wrigley T.V., Lim B.W., Hinman R.S. (2008). Role of muscle in the genesis and management of knee osteoarthritis. Rheum. Dis. Clin. N. Am..

[12] Rashid S.A., Hussain M.E., Bhati P., Veqar Z., Parveen A., Amin I., Rashid S.M. (2022). Muscle activation patterns around knee following neuromuscular training in patients with knee osteoarthritis: Secondary analysis of a randomized clinical trial. Arch. Physiother..

[13] Sattler L.N., Hing W.A., Vertullo C.J. (2019). What is the evidence to support early supervised exercise therapy after primary total knee replacement? A systematic review and meta-analysis. BMC Musculoskelet. Disord..

[14] Kim S.S., Lim K.T., Park J.W., Choi J.W., Yi C.H., Robinovitch S.N., Choi W.J. (2023). Effects of hip muscle activation on the stiffness and energy absorption of the trochanteric soft tissue during impact in sideways falls. J. Mech. Behav. Biomed. Mater..

[15] Piva S.R., Goodnite E.A., Childs J.D. (2005). Strength around the hip and flexibility of soft tissues in individuals with and without patellofemoral pain syndrome. J. Orthop. Sports Phys. Ther..

[16] Lightfoot A.P., Cooper R.G. (2016). The role of myokines in muscle health and disease. Curr. Opin. Rheumatol..

[17] Herrmann M., Engelke K., Ebert R., Müller-Deubert S., Rudert M., Ziouti F., Jundt F., Felsenberg D., Jakob F. (2020). Interactions between Muscle and Bone-Where Physics Meets Biology. Biomolecules.

[18] Barbalho S.M., Flato U.A.P., Tofano R.J., Goulart R.A., Guiguer E.L., Detregiachi C.R.P., Buchaim D.V., Araújo A.C., Buchaim R.L., Reina F.T.R. (2020). Physical Exercise and Myokines: Relationships with Sarcopenia and Cardiovascular Complications. Int. J. Mol. Sci..

[19] Mastrototaro L., Roden M. (2021). Insulin resistance and insulin sensitizing agents. Metabolism.

[20] Perényi H., Szegeczki V., Horváth G., Hinnah B., Tamás A., Radák Z., Ábrahám D., Zákány R., Reglodi D., Juhász T. (2020). Physical Activity Protects the Pathological Alterations of Alzheimer’s Disease Kidneys via the Activation of PACAP and BMP Signaling Pathways. Front. Cell Neurosci..

[21] Swift D.L., McGee J.E., Earnest C.P., Carlisle E., Nygard M., Johannsen N.M. (2018). The Effects of Exercise and Physical Activity on Weight Loss and Maintenance. Prog. Cardiovasc. Dis..

[22] Waller B., Munukka M., Rantalainen T., Lammentausta E., Nieminen M.T., Kiviranta I., Kautiainen H., Häkkinen A., Kujala U.M., Heinonen A. (2017). Effects of high intensity resistance aquatic training on body composition and walking speed in women with mild knee osteoarthritis: A 4-month RCT with 12-month follow-up. Osteoarthr. Cartil..

[23] Barone R., Szychlinska M.A. (2023). Highlights in Pathophysiology of the Musculoskeletal System. Int. J. Mol. Sci..

[24] Cianferotti L., Brandi M.L. (2014). Muscle-bone interactions: Basic and clinical aspects. Endocrine.

[25] Severinsen M.C.K., Pedersen B.K. (2020). Muscle-Organ Crosstalk: The Emerging Roles of Myokines. Endocr. Rev..

[26] Delezie J., Handschin C. (2018). Endocrine Crosstalk Between Skeletal Muscle and the Brain. Front. Neurol..

[27] Petridou A., Siopi A., Mougios V. (2019). Exercise in the management of obesity. Metabolism.

[28] Kanaley J.A., Colberg S.R., Corcoran M.H., Malin S.K., Rodriguez N.R., Crespo C.J., Kirwan J.P., Zierath J.R. (2022). Exercise/Physical Activity in Individuals with Type 2 Diabetes: A Consensus Statement from the American College of Sports Medicine. Med. Sci. Sports Exerc..

[29] Mahalakshmi B., Maurya N., Lee S.D., Bharath Kumar V. (2020). Possible Neuroprotective Mechanisms of Physical Exercise in Neurodegeneration. Int. J. Mol. Sci..

[30] Kong H., Wang X.Q., Zhang X.A. (2022). Exercise for Osteoarthritis: A Literature Review of Pathology and Mechanism. Front. Aging Neurosci..

[31] Dimitrios S. (2015). Exercise for tendinopathy. World J. Methodol..

[32] Bae S., Lee S., Park H., Ju Y., Min S.K., Cho J., Kim H., Ha Y.C., Rhee Y., Kim Y.P. (2023). Position Statement: Exercise Guidelines for Osteoporosis Management and Fall Prevention in Osteoporosis Patients. J. Bone Metab..

[33] Thomas R., Kenfield S.A., Yanagisawa Y., Newton R.U. (2021). Why exercise has a crucial role in cancer prevention, risk reduction and improved outcomes. Br. Med. Bull..

[34] Pedersen B.K. (2023). From the discovery of myokines to exercise as medicine. Dan. Med. J..

[35] Chen W., Wang L., You W., Shan T. (2021). Myokines mediate the cross talk between skeletal muscle and other organs. J. Cell Physiol..

[36] Leal L.G., Lopes M.A., Batista M.L. (2018). Physical Exercise-Induced Myokines and Muscle-Adipose Tissue Crosstalk: A Review of Current Knowledge and the Implications for Health and Metabolic Diseases. Front. Physiol..

[37] Boström P., Wu J., Jedrychowski M.P., Korde A., Ye L., Lo J.C., Rasbach K.A., Boström E.A., Choi J.H., Long J.Z. (2012). A PGC1-α-dependent myokine that drives brown-fat-like development of white fat and thermogenesis. Nature.

[38] Huh J.Y., Panagiotou G., Mougios V., Brinkoetter M., Vamvini M.T., Schneider B.E., Mantzoros C.S. (2012). FNDC5 and irisin in humans: I. Predictors of circulating concentrations in serum and plasma and, I.I. mRNA expression and circulating concentrations in response to weight loss and exercise. Metabolism.

[39] Huh J.Y., Siopi A., Mougios V., Park K.H., Mantzoros C.S. (2015). Irisin in response to exercise in humans with and without metabolic syndrome. J. Clin. Endocrinol. Metab..

[40] Huh J.Y., Mougios V., Kabasakalis A., Fatouros I., Siopi A., Douroudos I.I., Filippaios A., Panagiotou G., Park K.H., Mantzoros C.S. (2014). Exercise-induced irisin secretion is independent of age or fitness level and increased irisin may directly modulate muscle metabolism through AMPK activation. J. Clin. Endocrinol. Metab..

[41] Miyamoto-Mikami E., Sato K., Kurihara T., Hasegawa N., Fujie S., Fujita S., Sanada K., Hamaoka T., Tabata I., Iemitsu M. (2015). Endurance training-induced increase in circulating irisin levels is associated with reduction of abdominal visceral fat in middle-aged and older adults. PLoS ONE.

[42] Norheim F., Langleite T.M., Hjorth M., Holen T., Kielland A., Stadheim H.K., Gulseth H.L., Birkeland K.I., Jensen J., Drevon C.A. (2014). The effects of acute and chronic exercise on PGC-1α, irisin and browning of subcutaneous adipose tissue in humans. FEBS J..

[43] Pekkala S., Wiklund P.K., Hulmi J.J., Ahtiainen J.P., Horttanainen M., Pöllänen E., Mäkelä K.A., Kainulainen H., Häkkinen K., Nyman K. (2013). Are skeletal muscle FNDC5 gene expression and irisin release regulated by exercise and related to health?. J. Physiol..

[44] Roca-Rivada A., Castelao C., Senin L.L., Landrove M.O., Baltar J., Belén Crujeiras A., Seoane L.M., Casanueva F.F., Pardo M. (2013). FNDC5/irisin is not only a myokine but also an adipokine. PLoS ONE.

[45] Pardo M., Crujeiras A.B., Amil M., Aguera Z., Jiménez-Murcia S., Baños R., Botella C., de la Torre R., Estivill X., Fagundo A.B. (2014). Association of irisin with fat mass, resting energy expenditure, and daily activity in conditions of extreme body mass index. Int. J. Endocrinol..

[46] Li X., Zhu X., Wu H., Van Dyke T.E., Xu X., Morgan E.F., Fu W., Liu C., Tu Q., Huang D. (2021). Roles and Mechanisms of Irisin in Attenuating Pathological Features of Osteoarthritis. Front. Cell Dev. Biol..

[47] Ning K., Wang Z., Zhang X.A. (2022). Exercise-induced modulation of myokine irisin in bone and cartilage tissue-Positive effects on osteoarthritis: A narrative review. Front. Aging Neurosci..

[48] Tavassoli H., Heidarianpour A., Hedayati M. (2022). The effects of resistance exercise training followed by de-training on irisin and some metabolic parameters in type 2 diabetic rat model. Arch. Physiol. Biochem..

[49] Torabi A., Reisi J., Kargarfard M., Mansourian M. (2024). Differences in the Impact of Various Types of Exercise on Irisin Levels: A Systematic Review and Meta-Analysis. Int. J. Prev. Med..

[50] Vadalà G., Di Giacomo G., Ambrosio L., Cannata F., Cicione C., Papalia R., Denaro V. (2020). Irisin Recovers Osteoarthritic Chondrocytes In Vitro. Cells.

[51] Colaianni G., Sanesi L., Storlino G., Brunetti G., Colucci S., Grano M. (2019). Irisin and Bone: From Preclinical Studies to the Evaluation of Its Circulating Levels in Different Populations of Human Subjects. Cells.

[52] Kawao N., Iemura S., Kawaguchi M., Mizukami Y., Takafuji Y., Kaji H. (2021). Role of irisin in effects of chronic exercise on muscle and bone in ovariectomized mice. J. Bone Min. Metab..

[53] Shekhawat V.K., Hamilton J.L., Pacione C.A., Schmid T.M., Wimmer M.A. (2021). A Moving Contact of Articulation Enhances The Biosynthetic And Functional Responses of Articular Cartilage. Biotribology.

[54] Jia S., Yang Y., Bai Y., Wei Y., Zhang H., Tian Y., Liu J., Bai L. (2022). Mechanical Stimulation Protects Against Chondrocyte Pyroptosis Through Irisin-Induced Suppression of PI3K/Akt/NF-κB Signal Pathway in Osteoarthritis. Front. Cell Dev. Biol..

[55] Roggio F., Petrigna L., Trovato B., Di Rosa M., Musumeci G. (2023). The Role of Lubricin, Irisin and Exercise in the Prevention and Treatment of Osteoarthritis. Int. J. Mol. Sci..

[56] Zhu H., Liu H., Chen X., Xu X., Zhang S., Xie D. (2022). Enhancing autophagy and energy metabolism in the meniscus can delay the occurrence of PTOA in ACLT rat. Front. Cell Dev. Biol..

[57] Gong Z., Li J., He Z., Li S., Cao P., Ruan G., Zhang Y., Zeng Q., Chen R., Zheng P. (2022). Quadriceps strength is negatively associated with knee joint structural abnormalities-data from osteoarthritis initiative. BMC Musculoskelet. Disord..

[58] Chang J.S., Kim T.H., Nguyen T.T., Park K.S., Kim N., Kong I.D. (2017). Circulating irisin levels as a predictive biomarker for sarcopenia: A cross-sectional community-based study. Geriatr. Gerontol. Int..

[59] Park H.S., Kim H.C., Zhang D., Yeom H., Lim S.K. (2019). The novel myokine irisin: Clinical implications and potential role as a biomarker for sarcopenia in postmenopausal women. Endocrine..

[60] Baek J.Y., Jang I.Y., Jung H.W., Park S.J., Lee J.Y., Choi E., Lee Y.S., Lee E., Kim B.J. (2022). Serum irisin level is independent of sarcopenia and related muscle parameters in older adults. Exp. Gerontol..

[61] Guo M., Yao J., Li J., Zhang J., Wang D., Zuo H., Zhang Y., Xu B., Zhong Y., Shen F. (2023). Irisin ameliorates age-associated sarcopenia and metabolic dysfunction. J. Cachexia Sarcopenia Muscle.

[62] de Luis D., Primo D., Izaola O., Gómez J.J.L. (2024). Role of irisin and myostatin on sarcopenia in malnourished patients diagnosed with GLIM criteria. Nutrition.

[63] Demir L., Oflazoğlu U. (2023). The relationship between sarcopenia and serum irisin and TNF-α levels in newly diagnosed cancer patients. Support. Care Cancer.

[64] Oguz A., Sahin M., Tuzun D., Kurutas E.B., Ulgen C., Bozkus O., Gul K. (2021). Irisin is a predictor of sarcopenic obesity in type 2 diabetes mellitus: A cross-sectional study. Medicine.

[65] Lee Y.J., Heo Y., Choi J.H., Park S., Kim K.K., Shin D.W., Kang J.H. (2019). Association of Circulating Irisin Concentrations with Weight Loss after Roux-en-Y Gastric Bypass Surgery. Int. J. Environ. Res. Public Health.

[66] Fukushima Y., Kurose S., Shinno H., Thi Thu H.C., Takao N., Tsutsumi H., Hasegawa T., Nakajima T., Kimura Y. (2016). Effects of Body Weight Reduction on Serum Irisin and Metabolic Parameters in Obese Subjects. Diabetes Metab. J..

[67] Roberts L.D., Boström P., O’Sullivan J.F., Schinzel R.T., Lewis G.D., Dejam A., Lee Y.K., Palma M.J., Calhoun S., Georgiadi A. (2014). β-Aminoisobutyric acid induces browning of white fat and hepatic β-oxidation and is inversely correlated with cardiometabolic risk factors. Cell Metab..

[68] Jeremic N., Chaturvedi P., Tyagi S.C. (2017). Browning of White Fat: Novel Insight Into Factors, Mechanisms, and Therapeutics. J. Cell Physiol..

[69] Tanianskii D.A., Jarzebska N., Birkenfeld A.L., O’Sullivan J.F., Rodionov R.N. (2019). Beta-Aminoisobutyric Acid as a Novel Regulator of Carbohydrate and Lipid Metabolism. Nutrients.

[70] Jung T.W., Park H.S., Choi G.H., Kim D., Lee T. (2018). β-aminoisobutyric acid attenuates LPS-induced inflammation and insulin resistance in adipocytes through AMPK-mediated pathway. J. Biomed. Sci..

[71] Lyssikatos C., Wang Z., Liu Z., Warden S.J., Brotto M., Bonewald L. (2023). L-β-aminoisobutyric acid, L-BAIBA, a marker of bone mineral density and body mass index, and D-BAIBA of physical performance and age. Sci. Rep..

[72] Begriche K., Massart J., Abbey-Toby A., Igoudjil A., Lettéron P., Fromenty B. (2008). Beta-aminoisobutyric acid prevents diet-induced obesity in mice with partial leptin deficiency. Obesity.

[73] Faiz H., Malin S.K. (2023). A low-calorie diet raises β-aminoisobutyric acid in relation to glucose regulation and leptin independent of exercise in women with obesity. Front. Physiol..

[74] Kitase Y., Vallejo J.A., Gutheil W., Vemula H., Jähn K., Yi J., Zhou J., Brotto M., Bonewald L.F. (2018). β-aminoisobutyric Acid, l-BAIBA, Is a Muscle-Derived Osteocyte Survival Factor. Cell Rep..

[75] Seldin M.M., Peterson J.M., Byerly M.S., Wei Z., Wong G.W. (2012). Myonectin (CTRP15), a novel myokine that links skeletal muscle to systemic lipid homeostasis. J. Biol. Chem..

[76] Li Z., Yang Y.L., Zhu Y.J., Li C.G., Tang Y.Z., Ni C.L., Chen L.M., Niu W.Y. (2021). Circulating Serum Myonectin Levels in Obesity and Type 2 Diabetes Mellitus. Exp. Clin. Endocrinol. Diabetes.

[77] Li K., Liao X., Wang K., Mi Q., Zhang T., Jia Y., Xu X., Luo X., Zhang C., Liu H. (2018). Myonectin Predicts the Development of Type 2 Diabetes. J. Clin. Endocrinol. Metab..

[78] Mi Q., Li Y., Wang M., Yang G., Zhao X., Liu H., Zheng H., Li L. (2019). Circulating C1q/TNF-related protein isoform 15 is a marker for the presence of metabolic syndrome. Diabetes Metab. Res. Rev..

[79] Toloza F.J.K., Mantilla-Rivas J.O., Pérez-Matos M.C., Ricardo-Silgado M.L., Morales-Alvarez M.C., Pinzón-Cortés J.A., Pérez-Mayorga M., Arévalo-Garcia M.L., Tolosa-González G., Mendivil C.O. (2018). Plasma Levels of Myonectin But Not Myostatin or Fibroblast-Derived Growth Factor 21 Are Associated with Insulin Resistance in Adult Humans without Diabetes Mellitus. Front. Endocrinol..

[80] Peterson J.M., Wei Z., Wong G.W. (2009). CTRP8 and CTRP9B are novel proteins that hetero-oligomerize with C1q/TNF family members. Biochem. Biophys. Res. Commun..

[81] Seldin M.M., Tan S.Y., Wong G.W. (2014). Metabolic function of the CTRP family of hormones. Rev. Endocr. Metab. Disord..

[82] Kawaguchi M., Kawao N., Takafuji Y., Ishida M., Kaji H. (2020). Myonectin inhibits the differentiation of osteoblasts and osteoclasts in mouse cells. Heliyon.

[83] Li L., Wang Q., Qin C. (2020). Serum myonectin is increased after laparoscopic sleeve gastrectomy. Ann. Clin. Biochem..

[84] Butler A.E., Ramanjaneya M., Moin A.S.M., Hunt S.C., Atkin S.L. (2023). Clinical improvement may not reflect metabolic homeostasis normalization in subjects with and without Roux-En-Y bariatric surgery after 12 years: Comparison of surgical subjects to a lean cohort. Front. Endocrinol..

[85] Di Meo S., Iossa S., Venditti P. (2017). Skeletal muscle insulin resistance: Role of mitochondria and other ROS sources. J. Endocrinol..

[86] Gamas L., Matafome P., Seiça R. (2015). Irisin and Myonectin Regulation in the Insulin Resistant Muscle: Implications to Adipose Tissue: Muscle Crosstalk. J. Diabetes Res..

[87] Ozaki Y., Ohashi K., Otaka N., Kawanishi H., Takikawa T., Fang L., Takahara K., Tatsumi M., Ishihama S., Takefuji M. (2023). Myonectin protects against skeletal muscle dysfunction in male mice through activation of AMPK/PGC1α pathway. Nat. Commun..

[88] Otaka N., Shibata R., Ohashi K., Uemura Y., Kambara T., Enomoto T., Ogawa H., Ito M., Kawanishi H., Maruyama S. (2018). Myonectin Is an Exercise-Induced Myokine That Protects the Heart From Ischemia-Reperfusion Injury. Circ. Res..

[89] Nishii K., Aizu N., Yamada K. (2023). Review of the health-promoting effects of exercise and the involvement of myokines. Fujita Med. J..

[90] Pourranjbar M., Arabnejad N., Naderipour K., Rafie F. (2018). Effects of Aerobic Exercises on Serum Levels of Myonectin and Insulin Resistance in Obese and Overweight Women. J. Med. Life.

[91] Ji S., Park S.J., Lee J.Y., Baek J.Y., Jung H.W., Kim K., Yoo H.J., Jang I.Y., Kim B.J. (2023). Lack of association between serum myonectin levels and sarcopenia in older Asian adults. Exp. Gerontol..

[92] Lisco G., Disoteo O.E., De Tullio A., De Geronimo V., Giagulli V.A., Monzani F., Jirillo E., Cozzi R., Guastamacchia E., De Pergola G. (2024). Sarcopenia and Diabetes: A Detrimental Liaison of Advancing Age. Nutrients.

[93] Kharitonenkov A., Shiyanova T.L., Koester A., Ford A.M., Micanovic R., Galbreath E.J., Sandusky G.E., Hammond L.J., Moyers J.S., Owens R.A. (2005). FGF-21 as a novel metabolic regulator. J. Clin. Investig..

[94] Hojman P., Pedersen M., Nielsen A.R., Krogh-Madsen R., Yfanti C., Akerstrom T., Nielsen S., Pedersen B.K. (2009). Fibroblast growth factor-21 is induced in human skeletal muscles by hyperinsulinemia. Diabetes.

[95] Cuevas-Ramos D., Almeda-Valdés P., Meza-Arana C.E., Brito-Córdova G., Gómez-Pérez F.J., Mehta R., Oseguera-Moguel J., Aguilar-Salinas C.A. (2012). Exercise increases serum fibroblast growth factor 21 (FGF21) levels. PLoS ONE.

[96] Kim K.H., Kim S.H., Min Y.K., Yang H.M., Lee J.B., Lee M.S. (2013). Acute exercise induces FGF21 expression in mice and in healthy humans. PLoS ONE.

[97] Yang X., Karsenty G. (2004). ATF4, the osteoblast accumulation of which is determined post-translationally, can induce osteoblast-specific gene expression in non-osteoblastic cells. J. Biol. Chem..

[98] Lu H., Jia C., Wu D., Jin H., Lin Z., Pan J., Li X., Wang W. (2021). Fibroblast growth factor 21 (FGF21) alleviates senescence, apoptosis, and extracellular matrix degradation in osteoarthritis via the SIRT1-mTOR signaling pathway. Cell Death Dis..

[99] Yu Y., Li S., Liu Y., Tian G., Yuan Q., Bai F., Wang W., Zhang Z., Ren G., Zhang Y. (2015). Fibroblast growth factor 21 (FGF21) ameliorates collagen-induced arthritis through modulating oxidative stress and suppressing nuclear factor-kappa B pathway. Int. Immunopharmacol..

[100] Tillman E.J., Rolph T. (2020). FGF21: An Emerging Therapeutic Target for Non-Alcoholic Steatohepatitis and Related Metabolic Diseases. Front. Endocrinol..

[101] Le T.D.V., Fathi P., Watters A.B., Ellis B.J., Besing G.K., Bozadjieva-Kramer N., Perez M.B., Sullivan A.I., Rose J.P., Baggio L.L. (2023). Fibroblast growth factor-21 is required for weight loss induced by the glucagon-like peptide-1 receptor agonist liraglutide in male mice fed high carbohydrate diets. Mol. Metab..

[102] Jung H.W., Park J.H., Kim D.A., Jang I.Y., Park S.J., Lee J.Y., Lee S., Kim J.H., Yi H.S., Lee E. (2021). Association between serum FGF21 level and sarcopenia in older adults. Bone.

[103] Roh E., Hwang S.Y., Yoo H.J., Baik S.H., Cho B., Park Y.S., Kim H.J., Lee S.G., Kim B.J., Jang H.C. (2021). Association of plasma FGF21 levels with muscle mass and muscle strength in a national multicentre cohort study: Korean Frailty and Aging Cohort Study. Age Ageing.

[104] Tanaka T., Narazaki M., Kishimoto T. (2014). IL-6 in inflammation, immunity, and disease. Cold Spring Harb. Perspect. Biol..

[105] Muñoz-Cánoves P., Scheele C., Pedersen B.K., Serrano A.L. (2013). Interleukin-6 myokine signaling in skeletal muscle: A double-edged sword?. FEBS J..

[106] Nara H., Watanabe R. (2021). Anti-Inflammatory Effect of Muscle-Derived Interleukin-6 and Its Involvement in Lipid Metabolism. Int. J. Mol. Sci..

[107] Santos J.D.M.B.D., Bachi A.L.L., Luna Junior L.A., Foster R., Sierra A.P.R., Benetti M., Araújo J.R., Ghorayeb N., Kiss M.A.P.D.M., Vieira R.P. (2020). The Relationship of IL-8 and IL-10 Myokines and Performance in Male Marathon Runners Presenting Exercise-Induced Bronchoconstriction. Int. J. Environ. Res. Public. Health.

[108] Zou R., Li D., Wang G., Zhang M., Zhao Y., Yang Z. (2016). TAZ Activator Is Involved in IL-10-Mediated Muscle Responses in an Animal Model of Traumatic Brain Injury. Inflammation.

[109] Rose-John S. (2017). The Soluble Interleukin 6 Receptor: Advanced Therapeutic Options in Inflammation. Clin. Pharmacol. Ther..

[110] Raman A., Peiffer J.J., Hoyne G.F., Lawler N.G., Currie A.J., Fairchild T.J. (2018). Effect of exercise on acute postprandial glucose concentrations and interleukin-6 responses in sedentary and overweight males. Appl. Physiol. Nutr. Metab..

[111] Rochette E., Pascale D., Hourdé C., Evrard B., Pereira B., Echaubard S., Merlin E. (2018). Single Bout Exercise in Children with Juvenile Idiopathic Arthritis: Impact on Inflammatory Markers. Mediat. Inflamm..

[112] Nasi S., So A., Combes C., Daudon M., Busso N. (2016). Interleukin-6 and chondrocyte mineralisation act in tandem to promote experimental osteoarthritis. Ann. Rheum. Dis..

[113] Silacci P., Dayer J.M., Desgeorges A., Peter R., Manueddu C., Guerne P.A. (1998). Interleukin (IL)-6 and its soluble receptor induce TIMP-1 expression in synoviocytes and chondrocytes, and block IL-1-induced collagenolytic activity. J. Biol. Chem..

[114] Pedersen K.S., Dethlefsen C., Nielsen J., Gehl J., Pedersen B.K., Thor Straten P., Hojman P. (2016). Voluntary Running Suppresses Tumor Growth through Epinephrine- and IL-6-Dependent NK Cell Mobilization and Redistribution. Cell Metab..

[115] Ma Y., Gao M., Sun H., Liu D. (2015). Interleukin-6 gene transfer reverses body weight gain and fatty liver in obese mice. Biochim. Biophys. Acta (BBA) Mol. Basis Dis..

[116] Casimiro I., Hanlon E.C., White J., De Leon A., Ross R., Moise K., Piron M., Brady M.J. (2020). Reduction of IL-6 gene expression in human adipose tissue after sleeve gastrectomy surgery. Obes. Sci. Pract..

[117] Esposito K., Pontillo A., Di Palo C., Giugliano G., Masella M., Marfella R., Giugliano D. (2003). Effect of weight loss and lifestyle changes on vascular inflammatory markers in obese women: A randomized trial. JAMA.

[118] Patsalos O., Dalton B., Himmerich H. (2020). Effects of IL-6 Signaling Pathway Inhibition on Weight and BMI: A Systematic Review and Meta-Analysis. Int. J. Mol. Sci..

[119] Nash D., Hughes M.G., Butcher L., Aicheler R., Smith P., Cullen T., Webb R. (2023). IL-6 signaling in acute exercise and chronic training: Potential consequences for health and athletic performance. Scand. J. Med. Sci. Sports.

[120] Dalbeni A., Natola L.A., Garbin M., Zoncapè M., Cattazzo F., Mantovani A., Vella A., Canè S., Kassem J., Bevilacqua M. (2023). Interleukin-6: A New Marker of Advanced-Sarcopenic HCC Cirrhotic Patients. Cancers.

[121] Lin B., Bai L., Wang S., Lin H. (2021). The Association of Systemic Interleukin 6 and Interleukin 10 Levels with Sarcopenia in Elderly Patients with Chronic Obstructive Pulmonary Disease. Int. J. Gen. Med..

[122] Rong Y.D., Bian A.L., Hu H.Y., Ma Y., Zhou X.Z. (2018). Study on relationship between elderly sarcopenia and inflammatory cytokine IL-6, anti-inflammatory cytokine IL-10. BMC Geriatr..

[123] Reisinger K.W., Derikx J.P., van Vugt J.L., Von Meyenfeldt M.F., Hulsewé K.W., Olde Damink S.W., Stoot J.H., Poeze M. (2016). Sarcopenia is associated with an increased inflammatory response to surgery in colorectal cancer. Clin. Nutr..

[124] Pototschnig I., Feiler U., Diwoky C., Vesely P.W., Rauchenwald T., Paar M., Bakiri L., Pajed L., Hofer P., Kashofer K. (2023). Interleukin-6 initiates muscle- and adipose tissue wasting in a novel C57BL/6 model of cancer-associated cachexia. J. Cachexia Sarcopenia Muscle.

[125] Bilski J., Pierzchalski P., Szczepanik M., Bonior J., Zoladz J.A. (2022). Multifactorial Mechanism of Sarcopenia and Sarcopenic Obesity. Role of Physical Exercise, Microbiota and Myokines. Cells.

[126] Ferrucci L., Harris T.B., Guralnik J.M., Tracy R.P., Corti M.C., Cohen H.J., Penninx B., Pahor M., Wallace R., Havlik R.J. (1999). Serum IL-6 level and the development of disability in older persons. J. Am. Geriatr. Soc..

[127] Frost R.A., Lang C.H. (2007). Protein kinase B/Akt: A nexus of growth factor and cytokine signaling in determining muscle mass. J. Appl. Physiol..

[128] Moore D.R., Churchward-Venne T.A., Witard O., Breen L., Burd N.A., Tipton K.D., Phillips S.M. (2015). Protein ingestion to stimulate myofibrillar protein synthesis requires greater relative protein intakes in healthy older versus younger men. J. Gerontol. A Biol. Sci. Med. Sci..

[129] Kumar V., Atherton P.J., Selby A., Rankin D., Williams J., Smith K., Hiscock N., Rennie M.J. (2012). Muscle protein synthetic responses to exercise: Effects of age, volume, and intensity. J. Gerontol. A Biol. Sci. Med. Sci..

[130] Kalinkovich A., Livshits G. (2017). Sarcopenic obesity or obese sarcopenia: A cross talk between age-associated adipose tissue and skeletal muscle inflammation as a main mechanism of the pathogenesis. Ageing Res. Rev..

[131] Wang H., Wang N., Wang Y., Li H. (2022). Association between sarcopenia and osteoarthritis: A protocol for meta-analysis. PLoS ONE.

[132] Nadeau L., Aguer C. (2019). Interleukin-15 as a myokine: Mechanistic insight into its effect on skeletal muscle metabolism. Appl. Physiol. Nutr. Metab..

[133] Nielsen A.R., Mounier R., Plomgaard P., Mortensen O.H., Penkowa M., Speerschneider T., Pilegaard H., Pedersen B.K. (2007). Expression of interleukin-15 in human skeletal muscle effect of exercise and muscle fibre type composition. J. Physiol..

[134] Quinn L.S., Anderson B.G., Conner J.D., Wolden-Hanson T. (2013). IL-15 overexpression promotes endurance, oxidative energy metabolism, and muscle PPARδ, SIRT1, PGC-1α, and PGC-1β expression in male mice. Endocrinology.

[135] Quinn L.S., Anderson B.G., Conner J.D., Pistilli E.E., Wolden-Hanson T. (2011). Overexpression of interleukin-15 in mice promotes resistance to diet-induced obesity, increased insulin sensitivity, and markers of oxidative skeletal muscle metabolism. Int. J. Interferon Cytokine Mediat. Res..

[136] Furmanczyk P.S., Quinn L.S. (2003). Interleukin-15 increases myosin accretion in human skeletal myogenic cultures. Cell Biol. Int..

[137] Quinn L.S., Anderson B.G., Drivdahl R.H., Alvarez B., Argilés J.M. (2002). Overexpression of interleukin-15 induces skeletal muscle hypertrophy in vitro: Implications for treatment of muscle wasting disorders. Exp. Cell Res..

[138] Quinn L.S., Anderson B.G., Strait-Bodey L., Stroud A.M., Argilés J.M. (2009). Oversecretion of interleukin-15 from skeletal muscle reduces adiposity. Am. J. Physiol. Endocrinol. Metab..

[139] Khalafi M., Maleki A.H., Symonds M.E., Sakhaei M.H., Rosenkranz S.K., Ehsanifar M., Korivi M., Liu Y. (2024). Interleukin-15 responses to acute and chronic exercise in adults: A systematic review and meta-analysis. Front. Immunol..

[140] Crane J.D., MacNeil L.G., Lally J.S., Ford R.J., Bujak A.L., Brar I.K., Kemp B.E., Raha S., Steinberg G.R., Tarnopolsky M.A. (2015). Exercise-stimulated interleukin-15 is controlled by AMPK and regulates skin metabolism and aging. Aging Cell.

[141] Lan X.Q., Deng C.J., Wang Q.Q., Zhao L.M., Jiao B.W., Xiang Y. (2024). The role of TGF-β signaling in muscle atrophy, sarcopenia and cancer cachexia. Gen. Comp. Endocrinol..

[142] Joulia-Ekaza D., Cabello G. (2006). Myostatin regulation of muscle development: Molecular basis, natural mutations, physiopathological aspects. Exp. Cell Res..

[143] Baig M.H., Ahmad K., Moon J.S., Park S.Y., Ho Lim J., Chun H.J., Qadri A.F., Hwang Y.C., Jan A.T., Ahmad S.S. (2022). Myostatin and its Regulation: A Comprehensive Review of Myostatin Inhibiting Strategies. Front. Physiol..

[144] Rodgers B.D., Ward C.W. (2022). Myostatin/Activin Receptor Ligands in Muscle and the Development Status of Attenuating Drugs. Endocr. Rev..

[145] Stefanetti R.J., Voisin S., Russell A., Lamon S. (2018). Recent advances in understanding the role of FOXO3. F1000Res.

[146] Braun T., Gautel M. (2011). Transcriptional mechanisms regulating skeletal muscle differentiation, growth and homeostasis. Nat. Rev. Mol. Cell Biol..

[147] George I., Bish L.T., Kamalakkannan G., Petrilli C.M., Oz M.C., Naka Y., Sweeney H.L., Maybaum S. (2010). Myostatin activation in patients with advanced heart failure and after mechanical unloading. Eur. J. Heart Fail..

[148] Siff T., Parajuli P., Razzaque M.S., Atfi A. (2021). Cancer-Mediated Muscle Cachexia: Etiology and Clinical Management. Trends Endocrinol. Metab..

[149] Heineke J., Auger-Messier M., Xu J., Sargent M., York A., Welle S., Molkentin J.D. (2010). Genetic deletion of myostatin from the heart prevents skeletal muscle atrophy in heart failure. Circulation.

[150] Han H.Q., Zhou X., Mitch W.E., Goldberg A.L. (2013). Myostatin/activin pathway antagonism: Molecular basis and therapeutic potential. Int. J. Biochem. Cell Biol..

[151] Yang M., Liu C., Jiang N., Liu Y., Luo S., Li C., Zhao H., Han Y., Chen W., Li L. (2023). Myostatin: A potential therapeutic target for metabolic syndrome. Front. Endocrinol..

[152] Amor M., Itariu B.K., Moreno-Viedma V., Keindl M., Jürets A., Prager G., Langer F., Grablowitz V., Zeyda M., Stulnig T.M. (2019). Serum myostatin is upregulated in obesity and correlates with insulin resistance in humans. Exp. Clin. Endocrinol. Diabetes.

[153] Takao N., Kurose S., Miyauchi T., Onishi K., Tamanoi A., Tsuyuguchi R., Fujii A., Yoshiuchi S., Takahashi K., Tsutsumi H. (2021). The relationship between changes in serum myostatin and adiponectin levels in patients with obesity undergoing a weight loss program. BMC Endocr. Disord..

[154] Shan T., Liang X., Bi P., Kuang S. (2013). Myostatin knockout drives browning of white adipose tissue through activating the AMPK-PGC1α-Fndc5 pathway in muscle. FASEB J..

[155] Bueno P.G., Bassi D., Contrera D.G., Carnielli H.M., Silva R.N., Nonaka K.O., Selistre-de-Araújo H.S., Leal A.M.O. (2011). Post-exercise changes in myostatin and actRIIB expression in obese insulin-resistant rats. Mol. Cell. Endocrinol..

[156] Zhao C., Shao Y., Lin C., Zeng C., Fang H., Pan J., Cai D. (2017). Myostatin serum concentrations are correlated with the severity of knee osteoarthritis. J. Clin. Lab. Anal..

[157] Brightwell C.R., Latham C.M., Keeble A.R., Thomas N.T., Owen A.M., Reeves K.A., Long D.E., Patrick M., Gonzalez-Velez S., Abed V. (2023). GDF8 inhibition enhances musculoskeletal recovery and mitigates posttraumatic osteoarthritis following joint injury. Sci. Adv..

[158] Martin Gil C., Raoof R., Versteeg S., Willemen H.L.D.M., Lafeber F.P.J.G., Mastbergen S.C., Eijkelkamp N. (2024). Myostatin and CXCL11 promote nervous tissue macrophages to maintain osteoarthritis pain. Brain Behav. Immun..

[159] Scimeca M., Piccirilli E., Mastrangeli F., Rao C., Feola M., Orlandi A., Gasbarra E., Bonanno E., Tarantino U. (2017). Bone Morphogenetic Proteins and myostatin pathways: Key mediator of human sarcopenia. J. Transl. Med..

[160] Dechaumet B., Cleret D., Linossier M.T., Vanden-Bossche A., Chanon S., Lefai E., Laroche N., Lafage-Proust M.H., Vico L. (2020). Hypergravity as a gravitational therapy mitigates the effects of knee osteoarthritis on the musculoskeletal system in a murine model. PLoS ONE.

[161] Talbert E.E., Guttridge D.C. (2022). Emerging signaling mediators in the anorexia-cachexia syndrome of cancer. Trends Cancer..

[162] Lee S.J. (2021). Targeting the myostatin signaling pathway to treat muscle loss and metabolic dysfunction. J. Clin. Investig..

[163] Dodding M.P. (2017). Folliculin–A tumor suppressor at the intersection of metabolic signaling and membrane traffic. Small GTPases.

[164] Schiaffino S., Reggiani C. (2011). Fiber types in mammalian skeletal muscles. Physiol. Rev..

[165] Damer A., El Meniawy S., McPherson R., Wells G., Harper M.E., Dent R. (2022). Association of muscle fiber type with measures of obesity: A systematic review. Obes. Rev..

[166] Reyes N.L., Banks G.B., Tsang M., Margineantu D., Gu H., Djukovic D., Chan J., Torres M., Liggitt H.D., Hirenallur-S D.K. (2015). Fnip1 regulates skeletal muscle fiber type specification, fatigue resistance, and susceptibility to muscular dystrophy. Proc. Natl. Acad. Sci. USA.

[167] Zeng F., Cao J., Li W., Zhou Y., Yuan X. (2024). FNIP1: A key regulator of mitochondrial function. Biomed. Pharmacother..

[168] Xiao L., Yin Y., Sun Z., Liu J., Jia Y., Yang L., Mao Y., Peng S., Xie Z., Fang L. (2024). AMPK phosphorylation of FNIP1 (S220) controls mitochondrial function and muscle fuel utilization during exercise. Sci. Adv..

[169] Mao Y., Jin Z., Yang J., Xu D., Zhao L., Kiram A., Yin Y., Zhou D., Sun Z., Xiao L. (2024). Muscle-bone cross-talk through the FNIP1-TFEB-IGF2 axis is associated with bone metabolism in human and mouse. Sci. Transl. Med..

[170] Bliddal H., Bays H., Czernichow S., Uddén Hemmingsson J., Hjelmesæth J., Hoffmann Morville T., Koroleva A., Skov Neergaard J., Vélez Sánchez P., Wharton S. (2024). Once-Weekly Semaglutide in Persons with Obesity and Knee Osteoarthritis. N. Engl. J. Med..

